# Supplementary material for: Combining multiplexed assays of variant effect for enhanced BRCA2 variant classification
Source: Nat Commun. 2026 Apr 9;17:4847. doi: 10.1038/s41467-026-71393-0 (PMC13223280; doi:10.1038/s41467-026-71393-0)
Supplement: Supplementary file 1 — Supplementary Information [file 41467_2026_71393_MOESM1_ESM.pdf]

Combining multiplexed assays of variant effect for enhanced *BRCA2* variant classification

Supplementary Information

Supplementary Table 1: Functional categories of the six models ("Concordance model"; "Integrated VarCall model"; "Integrated GMM model"; "Secondary concordance model"; Huang et al., 2025; Sahu et al., 2025) by variant type.

Concordance model*		Canonical splice (N, %)		Intronic (N, %)		Missense (N, %)		Nonsense (N, %)		Silent (N, %)		Grand Total (N, %)	
	P strong	70	10.13	19	2.75	316	45.73	278	40.23	8	1.16	691	13.52
	P moderate	0	0.00	0	0.00	1	100.00	0	0.00	0	0.00	1	0.02
	P Str_mod	2	10.00	0	0.00	13	65.00	5	25.00	0	0.00	20	0.39
	P_mod_Str	0	0.00	1	3.45	26	89.66	2	6.90	0	0.00	29	0.57
	P_str_supp	1	12.50	0	0.00	5	62.50	2	25.00	0	0.00	8	0.16
	P_supp_str	0	0.00	2	12.50	13	81.25	1	6.25	0	0.00	16	0.31
	VUS	0	0.00	0	0.00	9	100.00	0	0.00	0	0.00	9	0.18
	B supporting	1	100.00	0	0.00	0	0.00	0	0.00	0	0.00	1	0.02
	B_supp_str	0	0.00	0	0.00	33	100.00	0	0.00	0	0.00	33	0.65
	B_str_supp	0	0.00	1	4.35	16	69.57	0	0.00	6	26.09	23	0.45
	B_mod_str	0	0.00	2	1.60	122	97.60	0	0.00	1	0.80	125	2.45
	B_str_mod	0	0.00	1	1.05	65	68.42	0	0.00	29	30.53	95	1.86
	B strong	2	0.05	105	2.59	2870	70.71	0	0.00	1082	26.66	4059	79.43
	Grand Total	76	1.49	131	2.56	3489	68.28	288	5.64	1126	22.04	5110	100.00
		Canonical splice (N, %)		Intronic (N, %)		Missense (N, %)		Nonsense (N, %)		Silent (N, %)		Grand Total (N, %)	
**Integrated VarCall model**	P strong	117	12.14	37	3.84	474	49.17	327	33.92	9	0.93	964	15.10
	P moderate	4	10.81	1	2.70	32	86.49	0	0.00	0	0.00	37	0.58
	P supporting	1	3.70	1	3.70	24	88.89	1	3.70	0	0.00	27	0.42
	VUS	4	5.80	6	8.70	57	82.61	1	1.45	1	1.45	69	1.08
	B supporting	1	3.13	1	3.13	30	93.75	0	0.00	0	0.00	32	0.50
	B moderate	2	2.41	5	6.02	74	89.16	0	0.00	2	2.41	83	1.30
	B strong	6	0.12	162	3.13	3747	72.46	0	0.00	1256	24.29	5171	81.01
	Grand Total	135	2.11	213	3.34	4438	69.53	329	5.15	1268	19.87	6383	100.00
		Canonical splice (N, %)		Intronic (N, %)		Missense (N, %)		Nonsense (N, %)		Silent (N, %)		Grand Total (N, %)	
**Integrated GMM model**	P strong	104	10.92	31	3.26	518	54.41	278	29.20	21	2.21	952	14.91
	P moderate	10	4.72	4	1.89	147	69.34	30	14.15	21	9.91	212	3.32
	P supporting	6	5.50	3	2.75	79	72.48	5	4.59	16	14.68	109	1.71
	VUS	7	2.49	10	3.56	225	80.07	6	2.14	33	11.74	281	4.40
	B supporting	2	1.23	9	5.52	115	70.55	2	1.23	35	21.47	163	2.55
	B moderate	0	0.00	21	5.04	318	76.26	4	0.96	74	17.75	417	6.53
	B strong	6	0.14	135	3.18	3036	71.45	4	0.09	1068	25.14	4249	66.57
	Grand Total	135	2.11	213	3.34	4438	69.53	329	5.15	1268	19.87	6383	100.00
**Secondary concordance model^**		Canonical splice (N, %)		Intronic (N, %)		Missense (N, %)		Nonsense (N, %)		Silent (N, %)		Grand Total (N, %)	
	P strong	102	12.52	27	3.31	399	48.96	278	34.11	9	1.10	815	14.19
	P moderate	1	16.67	0	0.00	5	83.33	0	0.00	0	0.00	6	0.10
	P_str_mod	8	9.20	3	3.45	46	52.87	30	34.48	0	0.00	87	1.51
	P_mod_str	1	7.14	1	7.14	12	85.71	0	0.00	0	0.00	14	0.24
	P _str_sup	1	5.00	2	10.00	12	60.00	5	25.00	0	0.00	20	0.35
	P_mod_sup	1	20.00	0	0.00	4	80.00	0	0.00	0	0.00	5	0.09
	P_sup_str	0	0.00	1	12.50	7	87.50	0	0.00	0	0.00	8	0.14
	P_sup_mod	0	0.00	0	0.00	7	100.00	0	0.00	0	0.00	7	0.12

	P supporting	0	0.00	0	0.00	2	100.00	0	0.00	0	0.00	2	0.03
	VUS	0	0.00	2	16.67	9	75.00	0	0.00	1	8.33	12	0.21
	B supporting	0	0.00	0	0.00	1	100.00	0	0.00	0	0.00	1	0.02
	B_sup_mod	0	0.00	1	33.33	2	66.67	0	0.00	0	0.00	3	0.05
	B_mod_sup	0	0.00	0	0.00	3	100.00	0	0.00	0	0.00	3	0.05
	B_str_sup	0	0.00	7	4.90	101	70.63	0	0.00	35	24.48	143	2.49
	B_sup_str	0	0.00	0	0.00	4	100.00	0	0.00	0	0.00	4	0.07
	B moderate	0	0.00	1	7.69	11	84.62	0	0.00	1	7.69	13	0.23
	B_mod_str	1	7.69	3	23.08	9	69.23	0	0.00	0	0.00	13	0.23
	B_str_mod	0	0.00	16	4.24	288	76.39	0	0.00	73	19.36	**377**	6.56
	B strong	4	0.09	130	3.09	3009	71.46	0	0.00	1068	25.36	4211	73.31
	Grand Total	119	2.07	194	3.38	3931	68.44	313	5.45	1187	20.67	5744	100.00
**Huang et al., Nature 2025**		Canonical splice (N, %)		Intronic (N, %)		Missense (N, %)		Nonsense (N, %)		Silent (N, %)		Grand Total (N, %)	
	P strong	119	11.66	50	4.90	502	49.17	339	33.20	11	1.08	1021	14.67
	P moderate	2	2.27	7	7.95	75	85.23	2	2.27	2	2.27	88	1.26
	P supporting	0	0.00	12	25.53	34	72.34	1	2.13	0	0.00	47	0.68
	VUS	4	3.28	22	18.03	93	76.23	0	0.00	3	2.46	122	1.75
	B supporting	1	1.64	8	13.11	52	85.25	0	0.00	0	0.00	61	0.88
	B moderate	3	1.58	18	9.47	166	87.37	0	0.00	3	1.58	190	2.73
	B strong	9	0.17	434	7.99	3661	67.42	0	0.00	1326	24.42	5430	78.03
	Grand Total	138	1.98	551	7.92	4583	65.86	342	4.91	1345	19.33	6959	100.00
**Sahu et al., Nature 2025**		Canonical splice (N, %)		Intronic (N, %)		Missense (N, %)		Nonsense (N, %)		Silent (N, %)		Grand Total (N, %)	
	P strong	77	6.92	38	3.42	651	58.54	282	25.36	64	5.76	1112	17.86
	P moderate	3	4.17	3	4.17	51	70.83	5	6.94	10	13.89	72	1.16
	P supporting	1	2.56	2	5.13	30	76.92	2	5.13	4	10.26	39	0.63
	VUS	6	1.75	7	2.04	275	80.17	8	2.33	47	13.70	343	5.51
	B supporting	1	3.45	1	3.45	21	72.41	0	0.00	6	20.69	29	0.47
	B moderate	1	0.93	3	2.78	75	69.44	0	0.00	29	26.85	108	1.73
	B strong	15	0.33	118	2.61	3277	72.47	13	0.29	1099	24.30	4522	72.64
	Grand Total	104	1.67	172	2.76	4380	70.36	310	4.98	1259	20.22	6225	100.00

P: pathogenic; B: benign; Str: strong; supp: supporting; mod: moderate; GMM: Gaussian mixture model

*: Concordance model is based on concordant results from Huang et al., Nature 2025 and Sohu et al., Nature 2025; when sub-categories were different (e.g. P str vs P mod), Huang et al., 2025 was listed before Sahu et al., 2025 and was seperated by a "_".

^: Secondary concordance model is based on concordant results from Integrated VarCall model and Integrated GMM model; when sub-categories were different (e.g. P str vs P mod), Integrated VarCall model was listed before Integrated GMM model and seperated by a "_".

Supplementary Table 2: Functional category comparison between the six models ("Concordance model"; "Integrated VarCall model"; "Integrated GMM model"; "Secondary concordance model"; Huang et al., 2025; Sahu et al., 2025).

		Integrated VarCall model (concordance: 95.3%*)							
		P strong	P moderate	P supporting	VUS	B supporting	B moderate	B strong	Grand Total
Huang et al., 2025	P strong	883	18	14	29	8	16	17	985
	P moderate	28	4	5	6	4	6	29	82
	P supporting	15	0	0	4	1	6	14	40
	VUS	15	6	1	3	2	8	63	98
	B supporting	4	1	1	5	4	1	38	54
	B moderate	12	3	2	2	1	7	141	168
	B strong	7	5	4	20	12	39	4869	4956
	Grand Total	964	37	27	69	32	83	5171	6383
		Integrated VarCall model (concordance: 86.4%*)							
		P strong	P moderate	P supporting	VUS	B supporting	B moderate	B strong	Grand Total
Sahu et al., 2025	P strong	765	16	6	23	16	36	234	1096
	P moderate	21	1	0	0	0	3	47	72
	P supporting	8	0	0	2	0	1	27	38
	VUS	41	11	5	7	3	9	264	340
	B supporting	1	0	1	0	1	0	25	28
	B moderate	5	2	1	2	1	1	96	108
	B strong	57	7	10	31	9	26	4329	4469
	#N/A^	66	0	4	4	2	7	149	232
	Grand Total	964	37	27	69	32	83	5171	6383
		Integrated GMM model (concordance: 87.7%*)							
		P strong	P moderate	P supporting	VUS	B supporting	B moderate	B strong	Grand Total
Huang et al., 2025	P strong	765	83	27	36	13	29	32	985
	P moderate	20	14	6	6	4	12	20	82
	P supporting	16	2	1	9	0	4	8	40
	VUS	15	10	4	12	6	16	35	98
	B supporting	11	3	4	7	3	4	22	54
	B moderate	18	13	3	15	8	24	87	168
	B strong	107	87	64	196	129	328	4045	4956
	Grand Total	952	212	109	281	163	417	4249	6383
		Integrated GMM model (concordance: 86.5%*)							
		P strong	P moderate	P supporting	VUS	B supporting	B moderate	B strong	Grand Total
Sahu et al., 2025	P strong	772	108	38	66	28	35	49	1096
	P moderate	20	6	5	7	9	9	16	72
	P supporting	5	4	2	8	1	4	14	38
	VUS	42	27	16	40	17	48	150	340
	B supporting	1	4	1	3	1	4	14	28
	B moderate	4	2	5	12	7	13	65	108
	B strong	34	54	35	131	96	287	3832	4469
	#N/A^	74	7	7	14	4	17	109	232
	Grand Total	952	212	109	281	163	417	4249	6383

Supplementary Table 3: Evaluation of the six models ("Concordance model"; "Integrated VarCall model"; "Integrated GMM model"; "Secondary concordance model"; Huang et al., Nature 2025; Sahu et al., Nature 2025) by comparing to common sets of ClinVar standards and HDR standards*.

ClinVar standards

Concordance model
(sensitivity[c]: 95.6%; specificity[d]: 97%; accuracy[e]: 96.2%)

	P strong	P Str_mod	P_supp_str	B supporting	B_str_supp	B_mod_str	B strong	Total
**P**	65	0	3	0	0	0	0	68
**LP**	22	1	0	0	0	0	0	23
**LB**	0	0	0	0	1	2	32	35
**B**	0	0	0	0	1	3	28	32
**Total**	87	1	3	0	2	5	60	158

Integrated VarCall model
(sensitivity: 98.9%; specificity: 100%; accuracy: 99.4%)

	P strong	P moderate	P supporting	VUS	B supporting	B moderate	B strong	Total
**P**	67	0	0	0	1	0	0	68
**LP**	23	0	0	0	0	0	0	23
**LB**	0	0	0	0	0	0	35	35
**B**	0	0	0	0	0	0	32	32
**Total**	90	0	0	0	1	0	67	158

Integrated GMM model
(sensitivity: 94.5%; specificity: 98.5%; accuracy: 96.2%)

	P strong	P moderate	P supporting	VUS	B supporting	B moderate	B strong	Total
**P**	64	3	0	0	0	1	0	68
**LP**	22	1	0	0	0	0	0	23
**LB**	0	0	0	0	0	2	33	35
**B**	0	0	0	0	1	1	30	32
**Total**	86	4	0	0	1	4	63	158

Secondary concordance model
(sensitivity: 94.5%; specificity: 98.5%; accuracy: 96.2%)

	P strong	P Str_mod	P_supp_str	B supp_mod	B_str_supp	B_str_mod	B strong	Total
**P**	64	3	0	1	0	0	0	68
**LP**	22	1	0	0	0	0	0	23
**LB**	0	0	0	0	0	2	33	35
**B**	0	0	0	0	1	1	30	32
**Total**	86	4	0	1	1	3	63	158

Huang et al., Nature 2025
(sensitivity: 96.7%; specificity: 100%; accuracy: 97.5%)

	P strong	P moderate	P supporting	VUS	B supporting	B moderate	B strong	Total
**P**	65	0	3	0	0	0	0	68
**LP**	23	0	0	0	0	0	0	23
**LB**	0	0	0	0	0	2	33	35
**B**	0	0	0	0	0	3	29	32
**Total**	88	0	3	0	0	5	62	158

Sahu et al., Nature 2025
(sensitivity: 98.9%; specificity: 97.1%; accuracy: 98.1%)

		P strong	P moderate	P supporting	VUS	B supporting	B moderate	B strong	Total
	**P**	68	0	0	0	0	0	0	68
	**LP**	22	1	0	0	0	0	0	23
	**LB**	0	0	0	0	1	0	34	35
	**B**	0	0	0	0	1	0	31	32
	**Total**	90	1	0	0	2	0	65	158
**HDR standards**		**Concordance model (sensitivity: 87%; specificity: 98.1%; accuracy: 94.6%)**							
		P strong	P Str_mod	P_supp_str	B_supp_str	B_str_supp	B_mod_str	B strong	Total
	**Abnormal**	87	7	4	0	0	0	2	100
	**Normal**	0	0	0	3	1	11	201	216
	**Total**	87	7	4	3	1	11	203	316
		**Integrated VarCall model (sensitivity: 97%; specificity: 100%; accuracy: 99.1%)**							
		P strong	P moderate	P supporting	VUS	B supporting	B moderate	B strong	Total
	**Abnormal**	97	0	0	0	1	0	2	100
	**Normal**	0	0	0	0	0	0	216	216
	**Total**	97	0	0	0	1	0	218	316
		**Integrated GMM model (sensitivity: 93%; specificity: 97.7%; accuracy: 96.2%)**							
		P strong	P moderate	P supporting	VUS	B supporting	B moderate	B strong	Total
	**Abnormal**	93	4	0	0	0	1	2	100
	**Normal**	0	0	0	0	5	9	202	216
	**Total**	93	4	0	0	5	10	204	316
		**Secondary concordance model (sensitivity: 93%; specificity: 97.7%; accuracy: 96.2%)**							
		P strong	P Str_mod	P_str_supp	B_sup_mod	B_str_sup	B_str_mod	B strong	Total
	**Abnormal**	93	4	0	1	0	0	2	100
	**Normal**	0	0	0	0	5	9	202	216
	**Total**	93	4	0	1	5	9	204	316
		**Huang et al., Nature 2025 (sensitivity: 89%; specificity: 98.6%; accuracy: 95.6%)**							
		P strong	P moderate	P supporting	VUS	B supporting	B moderate	B strong	Total
	**Abnormal**	89	6	3	0	0	0	2	100
	**Normal**	0	0	0	0	3	8	205	216
	**Total**	89	6	3	0	3	8	207	316
		**Sahu et al., Nature 2025 (sensitivity: 96%; specificity: 99.5%; accuracy: 98.4%)**							
		P strong	P moderate	P supporting	VUS	B supporting	B moderate	B strong	Total
	**Abnormal**	96	1	1	0	0	0	2	100
	**Normal**	0	0	0	0	1	3	212	216
	**Total**	96	1	1	0	1	3	214	316

[*]: ClinVar standards were variants (excluding nonsense and silent) from ClinVar trustworthy groups with consensus classifications; HDR standards were restricted to non-splicing missense variants. P: pathogenic; B: benign; Str: strong; supp: supporting; mod: moderate; HDR: Homology-Directed Repair.

[c]: Sensitivity is calculated by P strong functional category over ClinVar standards P/LP or HDR abnormal.

[d]: Specificity is calculated by B strong/moderate over ClinVar standards B/LB or HDR normal.

[e]: Accuracy is calculated by combined P strong and B strong/moderate functional categories over ClinVar standards or HDR.

Supplementary Table 4: Evaluation of the six models ("Concordance model"; "Integrated VarCall model"; "Integrated GMM model"; "Secondary concordance model"; Huang et al., Nature 2025; Sahu et al., Nature 2025) by comparing to individual set of ClinVar standards and HDR standards*.

ClinVar standards

Concordance model
(sensitivity[c]: 93.6%; specificity[d]: 95.8%; accuracy[e]: 94.5%)

	P strong	P_Str_mod	P_str_supp	VUS	B supporting	B_mod_str	B strong	Total
**P**	66	0	4	0	1	0	0	71
**LP**	22	1	0	0	0	0	0	23
**LB**	0	0	0	0	2	4	32	38
**B**	0	0	0	0	1	3	29	33
**Total**	88	1	4	0	4	7	61	**165**

Integrated VarCall model
(sensitivity: 91.7%; specificity: 94.2%; accuracy: 92.6%)

	P strong	P moderate	P supporting	VUS	B supporting	B moderate	B strong	Total
**P**	101	2	0	2	2	1	2	110
**LP**	31	1	0	1	0	0	1	34
**LB**	0	0	0	3	0	0	43	46
**B**	0	0	1	0	1	2	36	40
**Total**	132	3	1	6	3	3	82	**230**

Integrated GMM model
(sensitivity: 80.6%; specificity: 86%; accuracy: 82.6%)

	P strong	P moderate	P supporting	VUS	B supporting	B moderate	B strong	Total
**P**	88	7	5	7	0	2	1	110
**LP**	28	2	1	0	1	1	1	34
**LB**	0	1	1	4	0	3	37	46
**B**	1	2	1	1	1	2	32	40
**Total**	117	12	8	12	2	8	71	**230**

Secondary concordance model
(sensitivity: 87.6%; specificity: 96%; accuracy: 90.7%)

	P strong	P Str_mod	P_supp_str	VUS	B_str_supp	B_str_mod	B strong	Total
**P**	86	7	3	0	1	0	0	97
**LP**	27	3	1	0	0	0	1	32
**LB**	0	0	0	1	0	3	35	39
**B**	0	0	1	0	1	3	31	36
**Total**	113	10	5	1	2	6	67	**204**

Huang et al., Nature 2025
(sensitivity: 91.1%; specificity: 90%; accuracy: 90.5%)

	P strong	P moderate	P supporting	VUS	B supporting	B moderate	B strong	Total
**P**	102	1	3	3	1	1	1	112
**LP**	31	0	0	1	0	1	1	34
**LB**	2	3	0	6	2	5	87	105
**B**	1	1	0	0	0	3	40	45
**Total**	136	5	3	10	3	10	129	**296**

Sahu et al., Nature 2025
(sensitivity: 77.4%; specificity: 90.4%; accuracy: 82.6%)

	P strong	P moderate	P supporting	VUS	B supporting	B moderate	B strong	Total
**P**	72	0	1	8	1	1	10	93
**LP**	24	2	0	1	0	0	4	31
**LB**	1	1	0	0	1	2	40	45
**B**	1	0	0	2	2	0	33	38
**Total**	98	3	1	11	4	3	87	**207**

HDR standards

Concordance model
(sensitivity: 86.1%; specificity: 96.9%; accuracy: 93.6%)

	P strong	P Str_mod	P_str_supp	VUS	B_supp_str	B_str_mod	B strong	Total
**Abnormal**	87	8	4	0	0	0	2	101
**Normal**	0	0	0	1	6	12	207	226
**Total**	87	8	4	1	6	12	209	**327**

Integrated VarCall model
(sensitivity: 91%; specificity: 94.8%; accuracy: 93.6%)

	P strong	P moderate	P supporting	VUS	B supporting	B moderate	B strong	Total
**Abnormal**	111	2	0	3	1	1	4	122
**Normal**	5	2	2	3	3	3	269	287
**Total**	116	4	2	6	4	4	273	**409**

Integrated GMM model
(sensitivity: 86.1%; specificity: 86.4%; accuracy: 85.6%)

	P strong	P moderate	P supporting	VUS	B supporting	B moderate	B strong	Total
**Abnormal**	105	9	1	2	1	2	2	122
**Normal**	15	3	4	14	6	16	229	287
**Total**	120	12	5	16	7	18	231	**409**

Secondary concordance model
(sensitivity: 87.1%; specificity: 94.6%; accuracy: 92.2%)

	P strong	P Str_mod	P_str_supp	VUS	B_supp_str	B_str_mod	B strong	Total
**Abnormal**	101	9	1	1	2	0	2	116
**Normal**	4	2	2	1	5	17	227	258
**Total**	105	11	3	2	7	17	229	**374**

Huang et al., Nature 2025
(sensitivity: 84.6%; specificity: 91.5%; accuracy: 89.4%)

	P strong	P moderate	P supporting	VUS	B supporting	B moderate	B strong	Total
**Abnormal**	104	7	3	2	0	1	6	123
**Normal**	7	4	3	5	6	10	259	294
**Total**	111	11	6	7	6	11	265	**417**

Sahu et al., Nature 2025
(sensitivity: 84.9%; specificity: 85.1%; accuracy: 85%)

	P strong	P moderate	P supporting	VUS	B supporting	B moderate	B strong	Total
**Abnormal**	107	2	1	5	0	1	10	126
**Normal**	18	2	3	17	2	4	236	282
**Total**	125	4	4	22	2	5	246	**408**

[*]: ClinVar standards are variants (excluding nonsense and silent) from ClinVar trustworthy groups with consensus classifications; HDR restrict to non-splicing missense variants. P: pathogenic; B: benign; Str: strong; supp: supporting; mod: moderate; HDR: Homology-Directed Repair.

[c]: Sensitivity is calculated by P strong functional category over ClinVar standards P/LP or HDR abnormal.

[d]: Specificity is calculated by B strong/moderate over ClinVar standards B/LB or HDR normal.

[e]: Accuracy is calculated by combined P strong and B strong/moderate functional categories over ClinVar standards or HDR.

Supplementary Table 5: Final ACMG classification based on "Integrated VarCall model" by functional category within each variant type.

		Final ACMG classification						
		B	LB	LP	P	VUS	VUS (discordant*)	Grand Total
**Canonical splice**		0	3	13	113	3	3	135
	B moderate	0	0	0	0	2	0	2
	B strong	0	3	0	0	1	2	6
	B supporting	0	0	0	0	0	1	1
	P moderate	0	0	0	4	0	0	4
	P strong	0	0	12	105	0	0	117
	P supporting	0	0	0	1	0	0	1
	VUS	0	0	1	3	0	0	4
**Intronic**		2	161	34	1	15	0	213
	B moderate	1	0	0	0	4	0	5
	B strong	1	161	0	0	0	0	162
	B supporting	0	0	0	0	1	0	1
	P moderate	0	0	1	0	0	0	1
	P strong	0	0	33	1	3	0	37
	P supporting	0	0	0	0	1	0	1
	VUS	0	0	0	0	6	0	6
**Missense**		80	3689	223	14	391	41	4438
	B moderate	1	46	0	0	24	3	74
	B strong	78	3641	0	0	1	27	3747
	B supporting	1	1	0	0	26	2	30
	P moderate	0	0	1	0	29	2	32
	P strong	0	0	222	14	233	5	474
	P supporting	0	0	0	0	22	2	24
	VUS	0	1	0	0	56	0	57
**Nonsense**		0	0	0	329	0	0	329
	P strong	0	0	0	327	0	0	327
	P supporting	0	0	0	1	0	0	1
	VUS	0	0	0	1	0	0	1
**Silent**		33	1223	7	1	3	1	1268
	B moderate	0	0	0	0	2	0	2
	B strong	33	1223	0	0	0	0	1256
	P strong	0	0	7	1	0	1	9
	VUS	0	0	0	0	1	0	1
Total		115	5076	277	458	412	45	6383

*: Discordant refer to variants have conflicts between functional result and ClinVar standards or HDR data.
ACMG: American College of Medical Genetics; P: pathogenic; LP: likely pathogenic; B: benign; LB: likely pathogenic; VUS: variants of uncertain significance.

Supplementary Table 6: Associations between BRCA2 missense variants evaluated with the "Integrated VarCall Model" and breast and ovarian cancer[1]

	Case		Control		OR (95% CI)	P-value
	Number of variants	Number tested	Number of variants	Number tested		
**Breast cancer**						
Clinical testing collection (Ambry Genetics)[2] vs AllofUs[3]						
Missense P Str >=95% probability	347	301534	40	194610	6.46 (4.68-9.15)	$8.56x10^{-41}$
Missense P Str	363	301550	50	194610	5.46 (4.07-7.48)	$1.00X10^{-38}$
Missense P Mod	8	301195	0	194610	NA	NA
Missense P Sup	6	301193	4	194610	NA	NA
Missense VUS	17	301204	10	194610	1.09 (0.50-2.50)	0.84
Missense B Sup	3	301190	4	194610	NA	NA
Missense B Mod	9	301196	3	194610	NA	NA
Missense B Str	626	301813	578	194610	0.76 (0.68-0.86)	$9.06X10^{-6}$
ACMG P/LP	289	301476	28	194610	7.66 (5.26-11.59)	$1.41X10^{-37}$
ACMG B/LB	595	301782	554	194610	0.76 (0.67-0.85)	$7.76X10^{-6}$
Pathogenic missense standard[4]	156	306659	14	194610	7.33 (1.29-13.24)	$1.29X10^{-19}$
DBD Nonsense	245	301432	23	194610	8.43 (5.6-13.32)	$1.44X10^{-35}$
Benign missense standard[5]	178	72027	470	194610	1.08 (0.91-1.29)	0.38
Population-based breast cancer cohort[6]						
Missense P Str >=95% probability	82	81073	22	83247	3.83 (2.37-6.16)	$9.73x10^{-10}$
Missense P Str	89	81073	27	83247	3.39 (2.2-5.28)	$2.41X10^{-9}$
Missense P Mod	2	81073	3	83247	NA	NA
Missense P Sup	2	81073	0	83247	NA	NA
Missense VUS	0	81073	5	83247	NA	NA
Missense B Sup	141	81073	178	83247	0.81 (0.65-1.02)	0.12
Missense B Mod	3	81073	8	83247	NA	NA
Missense B Str	1459	81073	1525	83247	0.98 (0.91-1.06)	0.63
ACMG P/LP	58	81073	15	83247	3.97 (2.24-7.15)	$2.06X10^{-7}$
ACMG B/LB	1597	81073	1704	83247	0.96 (0.9-1.03)	0.27
Pathogenic missense standard	41	81073	8	83247	5.26 (2.46-12.4)	$9.15X10^{-7}$
DBD nonsense	79	81073	13	83247	6.24 (3.49-11.87)	$2.14X10^{-12}$
Benign missense standard	831	81073	909	83247	0.94 (0.85-1.03)	0.388
Clinical testing collection (Ambry Genetics) vs gnomADv4.1[7]						
Missense P Str >=95% probability	561	376778	43	181964	6.35 (4.67-8.90)	$4.09x10^{-29}$
Missense P Str	525	376778	49	181964	5.80 (4.36-7.92)	$2.84X10^{-30}$
Missense P Mod	8	301195	2	181964	NA	NA
Missense P Sup	6	301193	4	181964	NA	NA
Missense VUS	17	301204	4	181964	NA	NA
Missense B Sup	3	301190	1	181964	NA	NA
Missense B Mod	9	301196	1	181964	NA	NA
Missense B Str	751	364527	439	181964	0.85 (0.76-0.96)	0.01
ACMG P/LP	334	364110	14	181964	11.89 (7.26-21.27)	$9.58X10^{-20}$
ACMG B/LB	716	364492	422	181964	0.85 (0.75-0.96)	0.015
Pathogenic missense standard	185	376778	10	181964	8.73 (4.91-17.49)	$3.12X10^{-11}$

DBD nonsense	284	364060	25	181964	5.75 (3.9-8.9)	$1.43X10^{-16}$
Benign missense standard	229	88309	365	181964	1.24 (1.05-1.46)	0.01
**Ovarian cancer**						
Clinical testing collection (Ambry Genetics) vs AllofUs						
Missense P Str >=95% probability	52	32032	40	194610	7.82 (5.01-12.31)	$5.35x10^{-19}$
Missense P Str	55	32035	50	194610	6.83 (4.51-10.40)	$1.45X10^{-18}$
Missense P Mod	2	31982	0	194610	NA	NA
Missense P Sup	4	31981	1	194610	NA	NA
Missense VUS	2	31982	10	194610	NA	NA
Missense B Sup	0	31980	4	194610	NA	NA
Missense B Mod	1	31981	3	194610	NA	NA
Missense B Str	81	32061	578	194610	0.92 (0.72-1.17)	0.53
ACMG P/LP	39	32019	28	194610	8.11 (4.80-13.88)	$1.07X10^{-14}$
ACMG B/LB	78	32058	554	194610	0.93 (0.72-1.19)	0.58
Pathogenic missense standard	20	32606	14	194610	7.97 (3.86-16.86)	$3.11X10^{-8}$
DBD nonsense	51	32031	23	194610	12.74 (7.58-22.01)	$5.833X10^{-23}$
Benign missense standard	34	9598	470	194610	1.56 (1.08-2.19)	0.02
Clinical testing collection (Ambry Genetics) vs gnomADv4.1						
Missense P Str >=95% probability	88	40762	43	181964	9.56 (6.56-14.17)	$2.84x10^{-30}$
Missense P Str	82	40762	49	181964	8.64 (6.07-12.45)	$3.49X10^{-31}$
Missense P Mod	2	31980	2	181964	NA	NA
Missense P Sup	4	31980	4	181964	NA	NA
Missense VUS	2	31980	4	181964	NA	NA
Missense B Sup	0	31980	1	181964	NA	NA
Missense B Mod	1	31980	1	181964	NA	NA
Missense B Str	100	38761	439	181964	1.08 (0.86-1.32)	0.56
ACMG P/LP	51	38712	14	181964	16.95 (9.68-31.71)	$8.87X10^{-21}$
ACMG B/LB	96	38757	422	181964	1.08 (0.86-1.34)	0.49
Pathogenic missense standard	29	40762	10	181964	12.01 (6.15-25.28)	$1.16X10^{-11}$
DBD nonsense	57	38718	25	181964	10.86 (6.86-17.71)	$1.12X10^{-22}$
Benign missense standard	41	12384	365	181964	1.52 (1.08-2.07)	0.01

[1]: Ovarian cancer included malignant epithelial tumors of the ovary and fallopian tube; exclude variants with MAF>0.001%.
[2]: Clinical testing cohort collected cases from breast and ovarian cancer patients in whom genetic testing was undertaken at Ambry Genetics.
[3]: When use AllofUs non-cancer female as control, OR were calculated by using logistic regression adjusted for age and ancestry, two-sided.
[4]: Pathogenic missense standard included ENIGMA-designated missense pathogenic variants.
[5]: Benign missense standard included ENIGMA-designated missense benign variants.
[6]: Cases and controls from population-based cohort were from CARRIERS (Hu, et al. NEJM 2021) and BRIDGES (Breast Cancer Association Consortium, et al. NEJM 2021) studies, OR were calculated by Fisher's exact test, two-sided.
[7]: When use gnomAD v4.1 female (excluded UK Biobank) as control, OR were calculated by using weighted logistic regression with control populations weighted for the relative frequency of different races and ethnicities in the cases, two-sided.
OR: odds ratio; CI: confidence interval; P: Pathogenic; Str: strong; DBD: DNA binding domain; ENIGMA: Evidence-based Network for the Interpretation of Germline Mutant Alleles; NA: not applied (when either case or control # was less than 5 counts).

Supplementary Table 7: Bayesian posterior probabilities, likelihood ratio, points, and respective evidence strength calculated based on Bayesian interpretation of ACMG/AMP guidelines*.

Using prior 0.35	Posterior probability	Likelihood ratio	Points	Evidence strength^
	0.9583527	42.735005	4	P strong
	0.9	16.714286	4	P strong
	0.7787626	6.5372016	4	P strong
	0.5792549	2.5567952	2	P moderate
	0.4626542	1.5989982	1	P supporting
	0.2519166	0.6253916	-1	B supporting
	0.1739635	0.3911146	-2	B moderate
	0.0761005	0.1529707	-4	B strong
	0.0124432	0.0234	-4	B strong
Using prior 0.2	Posterior probability	Likelihood ratio	Points	Evidence strength
	0.9674451	118.86938	4	P strong
	0.9	36	4	P strong
	0.7315927	10.902724	4	P strong
	0.4521994	3.3019272	2	P moderate
	0.3123746	1.8171206	1	P supporting
	0.1209412	0.5503212	-1	B supporting
	0.0703843	0.3028534	-2	B moderate
	0.022416	0.0917202	-4	B strong
	0.0020987	0.0084126	-4	B strong
Using prior 0.1	Posterior probability	Likelihood ratio	Points	Evidence strength
	0.9749629	350.47	4	P strong
	0.9	81	4	P strong
	0.6753335	18.72	4	P strong
	0.3246665	4.33	2	P moderate
	0.1877318	2.08	1	P supporting
	0.050708	0.48	-1	B supporting
	0.0250371	0.23	-2	B moderate
	0.0059002	0.05	-4	B strong
	0.0003169	0	-4	B strong

*: Tavtigian et al. 2018 Genet Med

^: For functional assay, the points were capped at 4/-4 based on Clark et al, AJHG, 2022.

Supplementary Table 8: Comparison of functional categories between Integrated VarCall models applying prior probabillity of pathogenicity of 0.1 and 0.2.

		Integrated VarCall Model applying prior probability of pathogenicity of 0.2								Concordance[1]	Concordance[2]
		P strong	P moderate	P supporting	VUS	B supporting	B moderate	B strong	Total		
Integrated VarCall Model (prior probability of pathogenicity of 0.1)	P strong	922							922	98% (6256/6383)	99.5% (6351/6383)
	P moderate	42	17						59		
	P supporting		20	12					32		
	VUS			15	54	2			71		
	B supporting				15	21	4		40		
	B moderate					9	76	17	102		
	B strong						3	5154	5157		
	Grand Total	964	37	27	69	32	83	5171	6383		

[1]: Concordance was calculated as the sum of variants in the same individual functional category (P strong, P moderate, P supporting, VUS, B supporting, B moderate, B strong) divided by total number of variants evaluated in Integrated VarCall Model (n=6383).

[2]: Concordance was calculated as the sum of variants in the same broad functional category P (P strong, P moderate, P supporting), B (B strong, B moderate, B supporting), and VUS divided by total number of variants evaluated in Integrated VarCall model (n=6383).

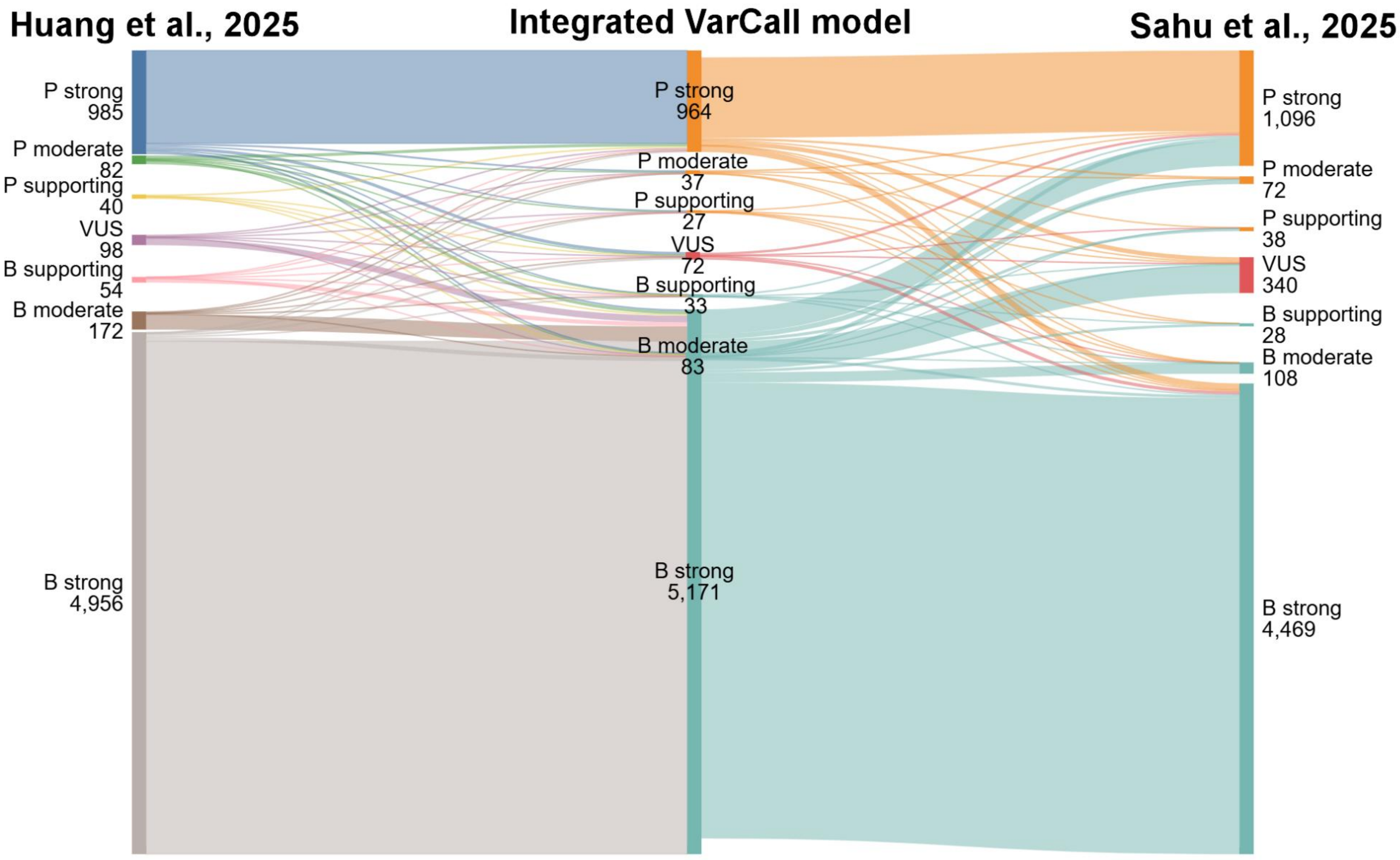

Supplementary Fig. 1: Comparison of Integrated VarCall model with the original MAVE studies. Sankey plot illustrates the functional category changes from Huang et al., 2025 (left) and Sahu et al., 2025 (right) to the Integrated VarCall model (middle).
