# Supplementary material for: Combining multiplexed assays of variant effect for enhanced BRCA2 variant classification
Source: Nat Commun. 2026 Apr 9;17:4847. doi: 10.1038/s41467-026-71393-0 (PMC13223280; doi:10.1038/s41467-026-71393-0)
Supplement: Supplementary file 3 — Reporting Summary [file 41467_2026_71393_MOESM3_ESM.pdf]

nature portfolio

Corresponding author(s): Fergus J. Couch
Last updated by author(s): 02/04/2026

Reporting Summary

Nature Portfolio wishes to improve the reproducibility of the work that we publish. This form provides structure for consistency and transparency in reporting. For further information on Nature Portfolio policies, see our Editorial Policies and the Editorial Policy Checklist.

Statistics

For all statistical analyses, confirm that the following items are present in the figure legend, table legend, main text, or Methods section.

n/a	Confirmed	
☐	☒	The exact sample size (*n*) for each experimental group/condition, given as a discrete number and unit of measurement
☐	☒	A statement on whether measurements were taken from distinct samples or whether the same sample was measured repeatedly
☐	☒	The statistical test(s) used AND whether they are one- or two-sided<br>*Only common tests should be described solely by name; describe more complex techniques in the Methods section.*
☐	☒	A description of all covariates tested
☐	☒	A description of any assumptions or corrections, such as tests of normality and adjustment for multiple comparisons
☐	☒	A full description of the statistical parameters including central tendency (e.g. means) or other basic estimates (e.g. regression coefficient) AND variation (e.g. standard deviation) or associated estimates of uncertainty (e.g. confidence intervals)
☐	☒	For null hypothesis testing, the test statistic (e.g. *F*, *t*, *r*) with confidence intervals, effect sizes, degrees of freedom and *P* value noted<br>*Give P values as exact values whenever suitable.*
☐	☒	For Bayesian analysis, information on the choice of priors and Markov chain Monte Carlo settings
☒	☐	For hierarchical and complex designs, identification of the appropriate level for tests and full reporting of outcomes
☐	☒	Estimates of effect sizes (e.g. Cohen's *d*, Pearson's *r*), indicating how they were calculated

Our web collection on statistics for biologists contains articles on many of the points above.

Software and code

Policy information about availability of computer code

Data collection	N/A
Data analysis	The custom code for functional score calculations was deposited in Github (najiemayo/Couch_SGE_BRCA2_MAVE_Mayo_NCI_joint_analysis)

For manuscripts utilizing custom algorithms or software that are central to the research but not yet described in published literature, software must be made available to editors and reviewers. We strongly encourage code deposition in a community repository (e.g. GitHub). See the Nature Portfolio guidelines for submitting code & software for further information.

Data

Policy information about availability of data

All manuscripts must include a data availability statement. This statement should provide the following information, where applicable:

- Accession codes, unique identifiers, or web links for publicly available datasets
- A description of any restrictions on data availability
- For clinical datasets or third party data, please ensure that the statement adheres to our policy

All data presented are available in the article and/or in GEO (GSE270424, GSE248438). All related code was deposited in Github (najiemayo/Couch_SGE_BRCA2_MAVE_Mayo_NCI_joint_analysis). The databases used in the study include GRCh38.

Research involving human participants, their data, or biological material

Policy information about studies with human participants or human data. See also policy information about sex, gender (identity/presentation), and sexual orientation and race, ethnicity and racism.

Reporting on sex and gender	Analyses were restricted to females because breast cancer is predominantly disease in women
Reporting on race, ethnicity, or other socially relevant groupings	Analyses were inclusive of all races and ethnicities. The numbers of cases and controls from sub-populations were insufficient for analyses.
Population characteristics	Breast cancer patients undergoing hereditary cancer testing that were selected using NCCN criteria to qualify for testing. GnomAD V4.1 and AllofUS public reference controls. Covariates include age or age at diagnosis of breast cancer, race/ethnicity, and personal/family history of cancer.
Recruitment	N/A
Ethics oversight	The analysis of the clinical-testing cohort was deemed exempt from review by the Western Institutional Review Board. The study was approved by Mayo Clinic IRB (15-007593).

Note that full information on the approval of the study protocol must also be provided in the manuscript.

Field-specific reporting

Please select the one below that is the best fit for your research. If you are not sure, read the appropriate sections before making your selection.

☒ Life sciences ☐ Behavioural & social sciences ☐ Ecological, evolutionary & environmental sciences

For a reference copy of the document with all sections, see nature.com/documents/nr-reporting-summary-flat.pdf

Life sciences study design

All studies must disclose on these points even when the disclosure is negative.

Sample size	No sample size calculations were used because this is an observational study.
Data exclusions	Common variants with frequencies in case or control populations of >0.001 were excluded.
Replication	Results from triplicate experiments from the Huang et al. study and duplicate experiments from the Sahu et al. study were combined.
Randomization	Samples and patients were not randomized. Data were organized by cancer case and control status.
Blinding	Blinding is not relevant to this study. Variant functional effects did not rely on any previous data. Statistical analyses were conducted based on case-control status.

Reporting for specific materials, systems and methods

We require information from authors about some types of materials, experimental systems and methods used in many studies. Here, indicate whether each material, system or method listed is relevant to your study. If you are not sure if a list item applies to your research, read the appropriate section before selecting a response.

Materials & experimental systems

n/a	Involved in the study	
☒	☐	Antibodies
☒	☐	Eukaryotic cell lines
☒	☐	Palaeontology and archaeology
☒	☐	Animals and other organisms
☒	☐	Clinical data
☒	☐	Dual use research of concern
☒	☐	Plants

Methods

n/a	Involved in the study	
☒	☐	ChIP-seq
☒	☐	Flow cytometry
☒	☐	MRI-based neuroimaging

Plants

Seed stocks	N/A
Novel plant genotypes	N/A
Authentication	N/A
