# Supplementary material for: Combining multiplexed assays of variant effect for enhanced BRCA2 variant classification
Source: Nat Commun. 2026 Apr 9;17:4847. doi: 10.1038/s41467-026-71393-0 (PMC13223280; doi:10.1038/s41467-026-71393-0)
Supplement: Supplementary file 4 — Description of Additional Supplementary Files [file 41467_2026_71393_MOESM4_ESM.pdf]

Description of Additional Supplementary Files

Supplementary Data 1: Variants evaluated in six models ("Concordance model"; "Integrated VarCall model"; "Integrated GMM model"; "Secondary concordance model"; Huang et al., 2025; Sahu et al., 2025).

Supplementary Data 2: ACMG classification of variants from "Integrated VarCall model".
